# The implementation and role of a staff naloxone program for non-profit community-based sites in British Columbia: A descriptive study

**DOI:** 10.1371/journal.pone.0251112

**Published:** 2021-05-13

**Authors:** Sierra Williams, Tanis King, Kristi Papamihali, Jane A. Buxton

**Affiliations:** 1 British Columbia Centre for Disease Control, Vancouver, BC, Canada; 2 University of British Columbia, Vancouver, BC, Canada; St. Michael’s Hospital, CANADA

## Abstract

**Introduction:**

The BC Centre for Disease Control implemented the Facility Overdose Response Box (FORB) program December 1^st^, 2016 to train and support non-healthcare service providers who may respond to an overdose in the workplace. The program aims to support staff at non-profit community-based organizations by ensuring policy development, training, practice overdose response exercises, and post-overdose debriefing opportunities are established and implemented.

**Materials and methods:**

Three data sources were used in this descriptive cross-sectional study: FORB site registration data; naloxone administration forms; and a survey that was distributed to FORB sites in February 2019. FORB program site and naloxone administration data from December 1^st^, 2016 to December 31^st^, 2019 were analyzed using descriptive statistics. A Cochran-Armitage test was used to assess trends over time in naloxone administration event characteristics. Site coordinator survey results are reported to supplement findings from administrative data.

**Results:**

As of December 31^st^, 2019, FORB was implemented at 613 sites across BC and 1,758 naloxone administration events were reported. The majority (86.3%, n = 1,517) were indicated as overdose reversals. At registration, 43.6% of sites provided housing services, 26.3% offered harm reduction supplies, and 18.6% provided Take Home Naloxone. Refusal to be transported to hospital following overdose events when emergency services were called showed an increasing trend over time. Most respondents (81.3%) reported feeling confident in their ability to respond to the overdose and 59.6% were offered staff debrief. Based on the 89 site survey responses, supports most commonly made available following an overdose were debrief with a fellow staff member (91.0%), debrief with a supervisor (89.9%), and/or counselling services (84.3%).

**Conclusions:**

The uptake of the FORB program has contributed to hundreds of overdose reversals in community settings in BC. Findings suggest that the FORB program supports developing staff preparedness and confidence in overdose response in community-based settings.

## Introduction

Rates of mortality and morbidity associated with opioid overdose continue to increase in North America as a result of a contaminated and unpredictable illicit drug supply [1, 2]. On April 14^th^, 2016, the provincial health officer of British Columbia (BC) declared a public health emergency in response to the unprecedented number of illicit drug overdose deaths in the province. Overdose fatalities now constitute the leading cause of unnatural deaths in BC and life expectancy at birth has declined [3, 4]. In June 2020, BC reported the highest number of drug toxicity deaths in one month to-date [3].

Naloxone, an opioid antagonist, is a cost-effective medication that can rapidly reverse an opioid overdose [5]. In Canada, most provinces and territories have implemented Take Home Naloxone (THN) programs with primary distribution through community sites and/or community pharmacies [6]. Community-based naloxone programs provide training and distribute THN kits so that individuals who may witness an overdose are prepared to recognize and respond; these programs have been found effective in reducing overdose deaths [7–9].

The BC THN program, which launched in 2012, was rapidly expanded in response to increasing overdose fatalities in 2016 [10–13]. Although the BC THN program was widely available across BC, the THN kits and supplies are not intended for occupational purposes. Consequently, staff at non-profit and community-based organizations which provide services for people who use substances (e.g. housing services and drop-in-centers), reported feeling inadequately prepared to respond to an overdose and articulated challenges with replacing used THN kits in a timely manner. To address these concerns, the BC Centre for Disease Control Harm Reduction Services (BCHRS) implemented the Facility Overdose Response Box (FORB) program on December 1^st^, 2016 in order to train and support non-healthcare service providers who may witness an overdose during the course of their work and ensure sufficient naloxone and other response supplies are available through centralized ordering processes [14, 15]. The program aims to support staff at non-profit and community-based organizations by ensuring program expectations of overdose response policy development, staff training, practice overdose response exercises (drills), and post-overdose debriefing opportunities are established and implemented at the site level.

The launch of FORB followed a successful pilot project involving two BC Health Authority Regions and a community-based organization in the Vancouver Mainland [16]. An evaluation of the FORB program was conducted from March to April 2018 through interviews with key stakeholders (BCHRS team members and regional harm reduction coordinators) to identify program successes and potential improvements [14]. As a result, a monthly FORB program infographic was created to highlight the FORB program and to distinguish it from the BC THN program, and program application and ordering forms were simplified [14, 15, 17].

To our knowledge, the FORB program is the first of its kind that directly aims to support non-healthcare service providers in settings where overdoses are likely to occur, including staff at housing services (e.g. emergency shelter and/or supportive or subsidized housing). The program addresses a limitation of existing provincial community-based naloxone programs where THN kits may not be suitable for use by staff in the workplace. Little evidence is available in the literature about programs such as FORB and the resources and supports needed by staff to feel prepared to respond to an overdose in non-healthcare settings.

The objective of this cross-sectional study is to describe the FORB program and its role in preparing community sites and non-healthcare service providers for overdose recognition and response. Specifically, we describe services provided by registered sites, characteristics of staff naloxone administration events, and staff preparedness for overdose response. The findings of this study can inform the implementation of staff naloxone programs for community-based sites in other jurisdictions both nationally and internationally.

## Materials and methods

### Setting

BCHRS works closely with Regional Health Authority Harm Reduction Coordinators who identify eligible organizations or sites and provide support in registration and initial staff training. Sites that are eligible to participate in the FORB program are non-profit and community-based organizations across BC that work with individuals at risk of experiencing an opioid overdose. Non-eligible sites include: government agencies, medical clinics and health authority sites, as well as private or for-profit businesses.

BCHRS operates the FORB program and is responsible for enrolling FORB sites, supplying overdose response boxes, naloxone, and replacement or training supplies to be kept at the site. BCHRS also develops training and program resources which are available on the program’s web-platform, Toward the Heart (www.towardtheheart.com) [18].

Prior to receiving overdose response boxes, sites are required to develop an overdose response policy and protocol for their organization and create a training plan for staff. Sites are responsible for training their staff to administer naloxone, providing support and debriefing to staff following an overdose response, and providing ongoing training and practice drills to maintain staff competency [14, 15].

### Study design

Three data sources were used in this descriptive cross-sectional study including: site registration data, naloxone administration forms, and a site coordinator survey.

For the primary analysis, we reviewed FORB program administrative data from December 1, 2016 to December 31, 2019. This includes site registration data and naloxone administration forms from 613 sites across BC. Naloxone administration forms, which are completed by staff responding to overdose events at FORB sites, collect demographic and overdose response information; responses without a date were excluded. For the secondary analysis, we used data from a survey that was distributed by e-mail to FORB site coordinators (e.g. an executive director or program coordinator) between February 7 to 22, 2019. The survey was piloted with BCHRS team members to assess face validity of questions.

### Study variables and analysis

All descriptive statistics were computed in Microsoft Excel and trend analyses were conducted in R version 3.6.3. For the site survey, ethics approval was obtained from the University of British Columbia Review Board (H12-02557) and consent to participate not applicable as data was analyzed anonymously.

Using FORB program administrative data, we executed a descriptive analysis using counts and proportions to outline the services offered at FORB sites, characteristics of naloxone administration events reported, and staff preparedness. We used site registration data to outline services offered by FORB sites.Variables related to naloxone administration events included: location of overdose, how overdose was recognized, first responder attendance, and if the client was transported to the hospital. We also assessed variables on reported staff confidence in overdose response, participation in debrief following overdose event, and number of overdoses responded to prior to the event in question.

Questions where participants did not respond were classified as missing and are provided for each variable. Additionally, a two-sided Cochran-Armitage test was used to assess if there were any trends over time in staff confidence in overdose response, overdose debrief, calling emergency services, naloxone administration by first responders, and transport to hospital after an overdose. Missing responses were excluded from trend analyses.

From the site coordinator survey, responses to questions on supports and training provided to staff were summarized and are shown in S1 File. Descriptive results from the site survey are reported to supplement findings from the FORB program administrative data on staff experiences with overdose response.

## Results

As of December 31, 2019, 613 sites across BC were registered in the FORB program. A total of 1,795 naloxone administration forms were received between December 1, 2016 to December 31, 2019. Thirty-seven respondents submitted forms without a precise date of naloxone administration and were excluded from the analysis, providing a final sample of 1,758. There were 89 respondents for the site coordinator survey.

### FORB site characteristics

Of the 613 sites registered, 471 (76.8%) of these sites were registered in the first 13 months (December 1, 2016 –December 31, 2017) of the program; while 87 (14.2%) and 55 (9.0%) sites joined in 2018 and 2019 respectively. Table 1 shows services offered at FORB sites based on the 613 site registration forms used in the analysis. At registration, nearly half (43.6%) of sites provided housing services, one quarter (26.3%) offered harm reduction supplies, 18.6% provided THN, and 3.3% were an overdose prevention service or supervised consumption site (OPS/SCS). OPS/SCS are locations where people can use previously obtained substances while being observed by individuals trained in overdose response [19].

**Table 1:** Services offered at FORB sites based on registration forms (n = 613).

Services offered^1^^,^^2^	n (%)
Drop in	158 (25.8)
Emergency shelter	76 (12.4)
Supportive housing	267 (43.6)
Subsidized housing	116 (18.9)
Counselling	139 (22.7)
Outreach services	178 (29.0)
Harm reduction supplies	161 (26.3)
Take home naloxone	114 (18.6)
Overdose Prevention Service/Supervised Consumption Site	20 (3.3)
Other	192 (31.3)

^1^ Based on registration forms submitted between December 1, 2016 –December 31, 2019.

^2^ Sites may provide more than one service and services provided by sites at registration may change over time.

### Characteristics of naloxone administration events

Of the 1,758 completed forms, 465 (26.5%) were completed in 2017, 595 (33.8%) in 2018 and 696 (39.6%) in 2019 (Table 2). The majority (86.3%, n = 1,517) of the naloxone administration events were indicated as overdose reversals, 1.3% (n = 23) were reported as overdose fatalities, and the remaining 12.4% (n = 218) were not specified. Among the 23 fatalities, 12 (52%) occurred in a private room, six (26%) were where staff responded offsite, three in a common area (13%) and two in a bathroom (9%). The majority of overdose events reported by FORB site staff were identified by someone yelling out for help (61.1%), while 18.9% involved the staff member witnessing the overdose first-hand (Table 2). Calling 911 was reported for most (81.8%) overdose events and among those, one-fifth (20.9%) had additional doses of naloxone administered by a first responder and less than half (44.0%) were transported to the hospital. A Cochran-Armitage test for trend did not show a change over time in calling 911 and administration of naloxone by first responders between 2017 and 2019, see the Table in S1 Appendix for details. As shown in the Table in S1 Appendix, refusal to be transported to hospital following overdose events where emergency services were called showed an increasing trend over time, with 46.7% of individuals refusing transport to hospital in 2017, 49.4% in 2018, and 53.4% in 2019 (p = 0.04).

**Table 2:** Characteristics of overdose reported through naloxone administration forms (n = 1,758) ^1^.

Overdose response characteristic	n (%)
**Naloxone administration events**	
2016	2 (0.1)
2017	465 (26.5)
2018	595 (33.8)
2019	696 (39.6)
**Location of overdose**	
Private room	512 (29.1)
Common area	492 (28.0)
Bathroom	138 (7.8)
Offsite–outside	430 (24.5)
Offsite–inside	17 (1.0)
Missing	169 (9.6)
**How did you know about the overdose**	
Someone yelled out for help	1074 (61.1)
Saw it happen	332 (18.9)
Scheduled (bath)room check	37 (2.1)
Random (bath)room check	63 (3.6)
Other	62 (3.5)
Missing	190 (10.8)
**911 called & first responder attended**	
Yes	1438 (81.8)
No	86 (4.9)
Missing	234 (13.3)
**First responder administered naloxone at the overdose scene**^**2**^	
Yes	301 (20.9)
Not needed	890 (61.9)
Missing	247 (17.2)
**Taken to hospital**^**2**^	
Yes	633 (44.0)
No–refused	645 (44.9)
No–other reason	65 (4.5)
Missing	95 (6.6)

^1^Based on completed and returned naloxone administration forms between December 1, 2016 –December 31, 2019.

^2^Limited to events where 911 was called and a first responder attended (n = 1,438).

### Staff preparedness and supports

#### Naloxone administration forms

Among the 1,758 overdose reversals reported by FORB sites, the vast majority of respondents (81.3%) reported feeling confident in their ability to respond to the overdose (Table 3). Table 3 shows most staff (59.6%) who responded to an overdose reported being offered debriefing; of these, 9.5% declined to participate. Almost half (45.4%) of the forms indicated that the staff member had responded to more than 10 overdoses (Table 3). A Cochran-Armitage test for trend did not show a change over time in staff confidence and participation in debrief between 2017 and 2019, see Table in S1 Appendix.

**Table 3:** Staff preparedness reported by naloxone administration forms (n = 1,758) ^1^.

Overdose response characteristics	n (%)
**Confident in responding to overdose**	
Yes	1429 (81.3)
Sort of	106 (6.0)
No	6 (0.3)
Not sure	27 (1.5)
Missing	190 (10.8)
**Participation in debrief after responding to an ov overdose**	
Yes	1047 (59.6)
No, was offered debrief but didn’t want	167 (9.5)
No, was not offered debrief	221 (12.6)
Not sure	134 (7.6)
Missing	189 (10.7)
**Number of overdoses responded to by staff**	
More than 10	798 (45.4)
6 to 10	282 (16.0)
2 to 5	337 (19.2)
Just this 1	96 (5.5)
Not sure	58 (3.3)
Missing	187 (10.6)

^**1**^Based on completed and returned naloxone administration forms between December 1, 2016 –December 31, 2019.

#### Survey data

The online survey was completed by 89 of 330 site coordinators from active FORB sites contacted, representing a response rate of 27%. Table in S2 Appendix shows that services offered at sites according to survey data include: harm reduction supplies, Take Home Naloxone, drop-in, emergency shelter, housing, counselling, outreach services, and OPS/SCS. Supports offered to staff members following responding to an overdose event are shown in Table 4. Debrief with a fellow staff member (91.0%), debrief with a supervisor (89.9%), and/or counselling services (84.3%) were the supports most commonly made available following an overdose event (Table 4). Table 4 also provides an overview of training characteristics including availability of new staff training and the frequency of refresher training and practice drills as reported by survey respondents. Most respondents (91.9%) indicated that sites provide ongoing training for new staff (Table 4). The majority of sites offered ongoing refresher training (89.5%) at least annually, whereas recurring practice drills were less common (61.6%) (Table 4). Among the site coordinators, the majority (n = 76, 86.0%) agreed that the FORB program helps staff respond to an overdose; 35 (39.8%) strongly agreed, 41 agreed (46.6%), and the remaining 12 (13.6%) reported being neutral.

**Table 4:** Supports offered to staff following an overdose event and ongoing staff training offered based on survey respondents.

Support offered	n (%)
**Debrief following overdose response**^**1**^	
Debrief with a fellow staff member	81 (91.0)
Debrief with a supervisor	80 (89.9)
Counselling services	75 (84.3)
Staff may take a break	68 (76.4)
Staff may leave the building	51 (57.3)
PHSA mobile response team	29 (32.6)
Other	6 (6.7)
**Training provided for new staff**^**2**^	
Yes	79 (91.9)
No	6 (7.0)
Not sure	1 (1.1)
**Frequency of refresher training**^**2**^	
Annually	30 (34.9)
Semi-annually	15 (17.4)
Quarterly	19 (22.1)
Bimonthly	5 (5.8)
Monthly	8 (9.3)
Biweekly/Weekly	0 (0)
Never	9 (10.5)
**Frequency of practice drills**^**2**^	
Annually	23 (26.7)
Semi-annually	11 (12.8)
Quarterly	8 (9.3)
Bimonthly	1 (1.2)
Monthly	7 (8.1)
Biweekly	2 (2.3)
Weekly	1 (1.2)
Never	33 (38.4)

^1^Based on total survey responses (n = 89).

^2^3 survey responses had missing data (n = 86).

## Discussion

There was a rapid uptake of the FORB program in BC, almost three-quarters of sites enrolled within the first year of program launch. FORB was introduced during a sudden and unprecedented increase in overdose deaths and coincided with the ramp up with the THN program and ministerial order for development of OPS sites in BC [9, 10, 20]. Upon enrollment, the majority of sites registered in the FORB program provided housing services which includes emergency shelter, supportive, and subsidized housing. FORB may also be implemented at housing service OPS sites. Respondents of the site survey were more likely to report offering THN and harm reduction supplies compared to services reported by sites at enrollment. This suggests sites may enrol with other BCHRS supply programs over time or that the sites that responded to the survey may be more engaged with the BCHRS programs. However, as the survey responses were anonymous, we are unable to determine the influence of these factors.

Previous program evaluation highlighted that the FORB program is easy to implement and sustain at all levels of the program and changes were implemented to help distinguish between the intended purpose of the BCHRS naloxone programs, as well as to improve application and supply management processes.^16^ Staff at FORB sites are trained to follow SAVE ME (stimulate, airway, ventilate, evaluate, medication, evaluate) protocols for overdose prevention, recognition, and response, which is consistent with training offered through the BC Take Home Naloxone program [9, 10, 21, 22]. Of staff who responded to an overdose, few reported lacking the confidence to respond to an overdose, and the high level of confidence was maintained over time. Our study found no significant change in overdose response, including calling 911 and administration of naloxone by first responders, as well as no change in staff confidence/debrief over time. This may be attributed to having clear organizational protocols, comprehensive and hands-on staff training, availability of BCHRS training resources, recurring drills, as well as frequent experience with responding to overdoses; all of which were previously highlighted as facilitators in the FORB program evaluation [15]. Having a variety of resources is important as site capacities, organizational structures, and training needs may vary.

The number of naloxone administration forms received each year has increased over time. This is consistent with increasing site numbers and overdose events but may also reflect completion of administration forms becoming a standard process. The vast majority of reported naloxone administration events were indicated as an overdose reversal. About half of the fatalities occurred in a private room and in one quarter, staff were called to respond to an event occurring off-site. It is plausible that the person was using drugs alone and/or that naloxone was not administered in time. This highlights the importance of messaging and initiatives around not using drugs alone and making access to observed consumption more readily available. For instance in 2020, initiatives including the downloadable Lifeguard Application and BeSafe Application were launched in BC to support connecting to emergency health services for individuals using drugs alone if an overdose occurred [23, 24]. In instances where staff did not specify the outcome, individuals may have been transported to the hospital and the long-term outcome was unknown when the form was submitted.

An increase in refusing transport to hospital was observed between 2017 and 2019. This is consistent with findings that have observed a notable decrease in transport across all regions in BC based on paramedic-attended overdose events [25]. Individuals at FORB sites may refuse transport to be monitored and supported by staff. Additional research is needed to understand why transport is declining and implications of non-transport as the reversal effect of naloxone can wear off and there may be adverse long-term outcomes such as brain injury, repeat overdose, or missed opportunities to offer interventions such as opioid agonist therapy [25].

Although site staff most often call 911 (81.8%), the majority of overdoses responded to by staff who report do not require additional naloxone administration by emergency health services while on site which may demonstrate timely overdose response by staff. Adherence to calling 911 is likely the result of embedding alerting 911 into the overdose response protocols at FORB sites. Frontline staff continue to have a critical role as first responders for overdose interventions. This has been demonstrated in settings such as OPS/SCS which provide observed consumption [26, 27], as well as at housing services [28] which are common FORB site types. Further research is needed to examine the importance of a staff naloxone program at locations where individuals may also be more likely to be using alone and observed consumption is not available such as at some housing services. Similarly, additional research should explore the role of staff naloxone programs in rural and remote areas where it may take additional time for emergency health services to arrive.

In light of the ongoing dual public health emergency with COVID-19, individuals may be at increased risk of overdose as the toxicity of the illicit drug supply is heightened [29]. Further, the capacity of sites that participate in FORB may be altered, housing services may implement restrictions around visitors, changes in practice for physical distancing, and individuals may be in isolation which increases risks of using alone. A program like FORB becomes even more relevant as it ensures an uninterrupted and dedicated supply of naloxone. The province has also experienced a decline in visits to OPS/SCS during the COVID-19 pandemic, which further increases risks of adverse outcomes from overdose [30]. Take Home Naloxone kits are widely available to community members throughout BC [12, 13], but the FORB program improves access to naloxone in BC by providing a dedicated supply of naloxone and protective equipment including CPR face shields, gloves, and staff training supplies for use by non-healthcare service providers in the workplace where there is a risk of overdoses on site [14, 15].This further eliminates challenges that may be encountered by community agencies that may have not have allocated budgets or capacities to source these supplies. Having these resources readily available helps non-healthcare service providers in being prepared and supported to respond to overdoses.

A program like FORB prompts community agencies to examine the policies and supports in place and ultimately the safety and well-being of their clients and staff. FORB site staff continue to report being offered debrief after response to an overdose. This is important as we learn more about the impacts of repeat overdose response for individuals who work at the frontline of the overdose crisis such as burnout, grief, stress and trauma [31–34]. At many of these community-based sites, staff may have close relationships and community connections; for example, staff at OPS are often peer responders [26, 32, 35]. This has implications for understanding the value of trauma-informed training, practice, and orienting mental health (trauma and grief) support services into these settings for staff [26, 32, 33]. In recognizing that individuals may have their own coping mechanisms, providing a range of supports will allow staff to choose the supports most appropriate for them after witnessing or responding to an overdose.

Our study has some limitations. Data regarding staff naloxone administration events depends on staff at FORB sites returning forms. Therefore, overdose events and reversals may be underreported as forms are not always submitted; this is minimised through the registration process which includes an agreement to send naloxone administration forms and is expected in order to receive replacement supplies. Although there is no follow-up with sites after forms are received, only 2% of forms were excluded due to a missing date. Data collection is intended to be low-barrier, however site names are indicated; there is potential for under-reporting of overdose events with adverse outcomes. As these forms are completed by individuals, self-reported data is susceptible to response bias.

The anonymous online survey had a response rate of 27%, thus results cannot be generalised to all regions and types of FORB sites in BC; however, respondents identified their sites provided a wide range of services. Survey respondents may be more engaged with BCHRS. The findings are based on site coordinators responses which may differ from the perceptions of frontline staff and may be subject to response bias.

## Conclusion

The BC FORB program is one of many innovative responses to the illicit drug crisis in BC and has contributed to hundreds of overdose reversals in community settings. Our findings suggest that FORB supports non-healthcare service providers in being confident and competent in recognizing and responding to an overdose on site and prompts sites to have a variety of debrief supports available. Implementation of programs similar to FORB that support staff at community-based and non-profit sites where overdoses are likely to occur should be considered in other jurisdictions nationally and internationally. Additional research is needed to explore considerations around staff debriefing, perceptions of clients at participating FORB sites, as well as variations in uptake and management of the program in rural and remote areas.

## Supporting information

S1 File FORB quantitative site survey. (DOCX)

S1 Appendix Characteristics of naloxone administration events at FORB sites over time, 2017–2019 (n = 1,756). (DOCX)

S2 Appendix Services offered at FORB sites based on survey data (n = 89). (DOCX)
