# Supplementary material for: The implementation and role of a staff naloxone program for non-profit community-based sites in British Columbia: A descriptive study
Source: PLoS One. 2021 May 13;16(5):e0251112. doi: 10.1371/journal.pone.0251112 (PMC8118334; doi:10.1371/journal.pone.0251112)
Supplement: S1 File — (DOCX) [file pone.0251112.s001.docx]

**BC Facility Overdose Response Box (FORB) Site Evaluation Survey**

***Page 2***

***1.The following section will outline your site(s) background and involvement as a FORB site.***

**a)When did you become a FORB site(s)?**

More than 1 year

Between 6 months – 1 year

Less than 6 months

Not sure

**b)What is your position?**

Executive Director

Site Coordinator

Other

**c)Did your site(s) have naloxone before becoming a FORB site?**

Yes

No

**d)Did your site(s) have a formal opioid overdose response policy and/or protocol prior to being a FORB site?**

Yes

No

Not sure

**If yes, did policy and/or protocol change after becoming a FORB site?**

Yes

No

**If no, how long did it take to develop an overdose response policy and/or protocol when becoming a FORB site? (In weeks): ______________________**

**e)What services are primarily offered at your site(s)? Please check all that apply.**

Supportive housing

Outreach services

Subsidized housing

Drop in

Counselling

Harm reduction supplies

Shelter

Take home naloxone

Observed injection space

**f)In the past 6 months, how often did an overdose occur at your site(s)?**

Less than 1 per month

1-3 per month

1 per week

2-6 per week

1 per day

Less than 1 per day

**g)Where are overdose most commonly occurring at your site?**

Common living area

Shared washroom

Individual resident room

Observed injection space

Other

Not applicable

**h)When do overdose events most frequently occur at your site? (select all that apply)**

Morning

Afternoon

Evening

Night

**i)Many overdose deaths are occurring in people who use alone. Devices and phone applications are being developed to monitor a person using drugs alone (i.e. in an individual room) which could alert staff if a person has an overdose.**

*Devices which monitor breathing and alert staff when breathing slows or stops: 
1) a wall mounted or table top device directed toward a client, 
2) a smartphone application directed toward a client from a table top,*

*Use of phone: 
3) applications such as face time to monitor the client after drug use 
4) client informing staff about use and requires a text/phone to confirm conscious*

***Do you think your site would consider implementing any of these initiatives?***

Yes

No

Not sure

**j)Please answer the following questions regarding the potential use of these monitoring devices:
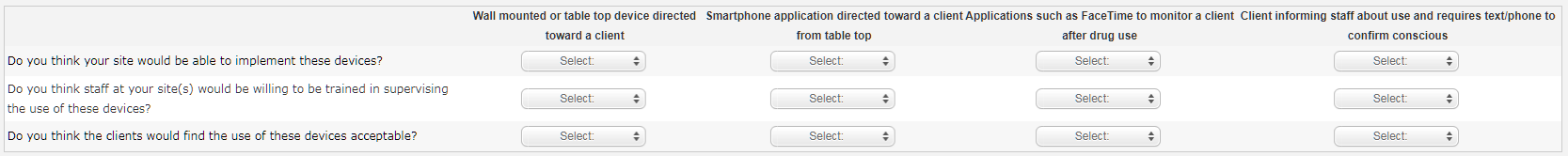
**

Yes

No

Not sure

***Page 3***

***2.The following questions will assess the usefulness of the FORB checklists. Please indicate your level of agreement with the following statements:***

1=strongly disagree, 2=disagree, 3=neutral, 4=agree, 5=strongly agree

**a)The checklist was easy to use and understand**

**
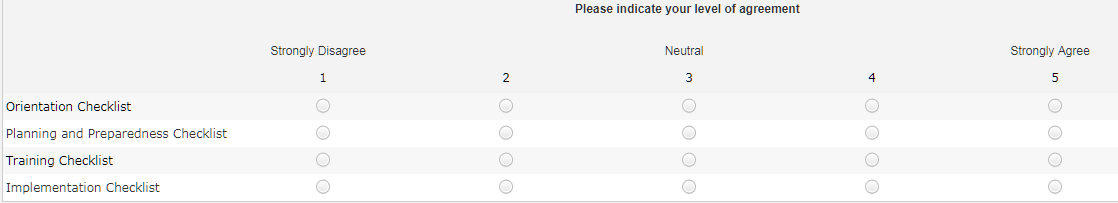
**

**b)The checklist provided all necessary information and resources.**

**
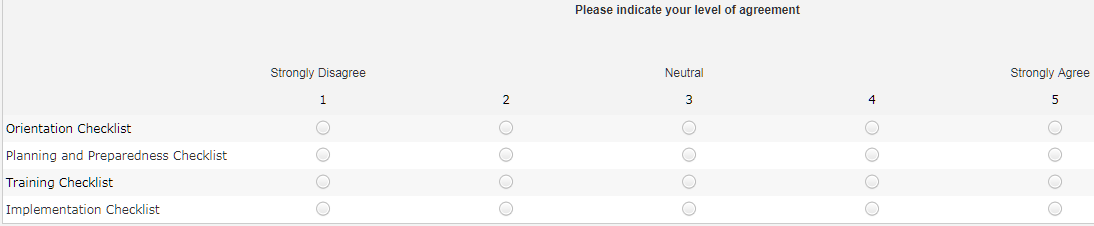
**

**Please explain anything that was missing or could be removed from any of the checklists:_____________**

***Page 4***

***3.This section will evaluate the effectiveness of FORB resources.***

**a)Please indicate your level of agreement with the following statements:**

*Please leave blank any statements that are not applicable.*


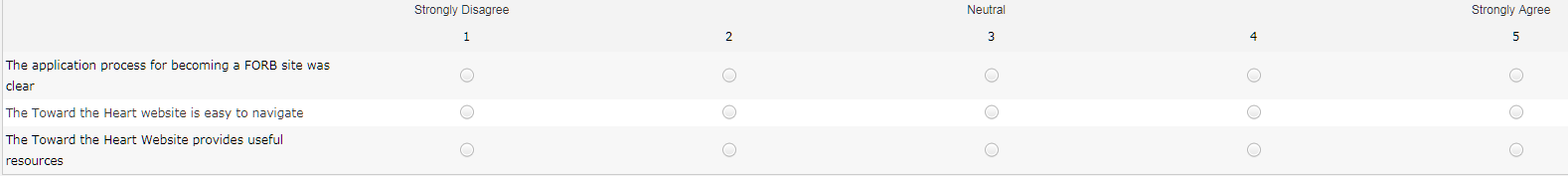


**b)The FORB box arrived with all the necessary supplies.**

Yes

No

Not sure

**c)Has your site(s) ordered subsequent supplies?**

Yes

No

Not sure

**If yes, have the subsequent supplies arrived within a timely manner?**

1-Never

2-

3-Sometimes

4-

5-Always

**d)How would you rate the helpfulness of the BC harm reduction program team?**

1-Not at all helpful

2-

3-Somewhat helpful

4-

5-Extremely helpful

**e)Did your site(s) experience confusion between the FORB program and the take home naloxone program?**

Yes

No

**f)If you answered yes to the previous question, please explain:________________**

***Page 5***

***4.This section will assess the implementation of training and ongoing activities as a FORB site.***

**a)How many staff could be trained at your site/organization**

0

1 to 10

11 to 20

21 to 30

31 to 40

41 to 50

**b)How many staff have been training at your site/organization?_________**

**c)How long did your site take to train all staff?**

< 1 month

1 month

2-3 months

4-6 months

7-12 months

>1 year

**d)How many trained staff are on site each shift?**

Daytime: select 1 / 2 / 3 / 4 / 5 / >5

Nighttime: select 1 / 2 / 3 / 4 / 5 / >5

**e)Did your site use training resources provided by the BC harm reduction program?**

Yes

No

Not sure

**f)What materials and activities were used to provide training to staff? Please check all that apply.**

Online training tool

Quick learn lesson

Naloxone training manual

Training checklist

Training videos

SAVE ME poster

How to respond to an opioid overdose poster

Other: _________

**g)Are training sessions ongoing for new staff?**

Yes

No

Not sure

**h)How often do you offer refresher training?**

Weekly

Biweekly

Monthly

Bimonthly

Quarterly

Semi-annually

Annually

Never

**Do you plan to provide refresher training in the future?**

Yes

No

**i)How often do you complete practice drills?**

Weekly

Biweekly

Monthly

Bimonthly

Quarterly

Semi-annually

Annually

Never

**Do you plan complete practice drills in the future?**

Yes

No

**j)Do you feel like you have the resources available to continue with staff training?**

1-Strongly disagree

2-

3-Neutral

4-

5-Strongly agree

**k)Please outline below any additional resources that may be useful to support your site:____________**

**l)What types of support are provided to staff following responding to an overdose?**

*Please check all that apply.*

Staff may take a break

Staff may leave the building

Debrief with a fellow staff member

Debrief with supervisor

Counselling services available

Use the PHSA mobile response team

Other

None of the above

***Page 6***

***5.This final section will assess your overall satisfaction with the FORB program. Please indicate your level of agreement with the following statements.***

**a)Staff are well prepared to recognize and respond to an overdose event.**

1-Never

2-

3-Sometimes

4-

5-Always

**b)The FORB program has helped your site be prepared to respond to an overdose event.**

1-Strongly disagree

2-

3-Neutral

4-

5-Strongly agree

**c)Overall, I’m satisfied with the services provided by the BC harm reduction program and with being a FORB site.**

1-Strongly disagree

2-

3-Neutral

4-

5-Strongly agree
