# Supplementary material for: The implementation and role of a staff naloxone program for non-profit community-based sites in British Columbia: A descriptive study
Source: PLoS One. 2021 May 13;16(5):e0251112. doi: 10.1371/journal.pone.0251112 (PMC8118334; doi:10.1371/journal.pone.0251112)
Supplement: S1 Appendix — (DOCX) [file pone.0251112.s002.docx]

**S1 Appendix. Characteristics of naloxone administration events at FORB sites over time, 2017 – 2019 (n=1,756).**

|  | **2017**  **(n=465)** | **2018**  **(n=595)** | **2019**  **(n=696)** | **Total^1^**  **(n=1,756)** | ***p* value^2^** |
| --- | --- | --- | --- | --- | --- |
| **Overdose response characteristics** | **n (%)** | **n (%)** | **n (%)** | **n (%)** |  |
| **Staff confident in responding to overdose** |  |  |  |  | 0.86 |
| Yes | 336 (94.1) | 470 (86.9) | 621 (93.0) | 1,427 (91.1) |  |
| Sort of/No/Not sure | 21 (5.9) | 71 (13.1) | 47 (7.0) | 139 (8.9) |  |
| **Staff participation in debrief after responding to overdose** |  |  |  |  | 0.30 |
| Yes | 232 (75.3) | 343 (67.7) | 472 (76.4) | 1,047 (73.1) |  |
| No | 76 (24.7) | 164 (32.3) | 146 (23.6) | 386 (26.9) |  |
| **911 called & first responder attended** |  |  |  |  | 0.46 |
| Yes | 317 (93.0) | 509 (94.4) | 610 (93.2) | 1,436 (94.3) |  |
| No | 24 (7.0) | 26 (5.6) | 36 (6.8) | 86 (5.7) |  |
| **First responder administered naloxone^3^** |  |  |  |  | 0.26 |
| Yes | 62 (26.4) | 116 (27.2) | 123 (23.3) | 301 (25.3) |  |
| No | 173 (73.6) | 311 (72.8) | 404 (76.7) | 888 (74.7) |  |
| **Taken to hospital^3^** |  |  |  |  | 0.04 |
| Yes | 149 (53.3) | 225 (50.6) | 259 (46.6) | 633 (49.6) |  |
| No | 128 (46.7) | 218 (49.4) | 297 (53.4) | 643 (50.4) |  |

*Trend analyses were limited to naloxone administration forms completed and returned between January 1, 2017 – December 31, 2019. Two records from 2016 were excluded from analyses.*

*^1^ Naloxone administration forms with missing data were excluded from trend analyses and account for differences in sample size for each variable assessed*

*^2^ p value reflects significance of Cochran-Armitage test for trend*

*^3^ Limited to events where 911 was called and a first responder attended (n=1,436)*
