# Supplementary material for: The implementation and role of a staff naloxone program for non-profit community-based sites in British Columbia: A descriptive study
Source: PLoS One. 2021 May 13;16(5):e0251112. doi: 10.1371/journal.pone.0251112 (PMC8118334; doi:10.1371/journal.pone.0251112)
Supplement: S2 Appendix — (DOCX) [file pone.0251112.s003.docx]

**S2 Appendix. Services offered at FORB sites based on survey data (n=89)^1^**

| **Services offered at site according to survey data^2^** | **n (%)** |
| --- | --- |
| Drop in | 43 (48.3) |
| Emergency shelter | 25 (28.1) |
| Supportive housing | 52 (58.4) |
| Subsidized housing | 21 (23.6) |
| Counselling | 35 (39.3) |
| Outreach services | 52 (58.4) |
| Harm reduction supplies | 38 (42.7) |
| Take home naloxone | 37 (41.6) |
| Observed consumption site^5^ | 15 (16.9) |

*^1^ 89 is based on number of respondents from quantitative site survey administered February 2019
^2^ Sites may provide more than one service and services provided by sites at registration may change over time*
